# Motivation 2 Quit (M2Q): A cluster randomized controlled trial evaluating the effectiveness of Tobacco Cessation on Prescription in Swedish primary healthcare

**DOI:** 10.1371/journal.pone.0278369

**Published:** 2022-12-01

**Authors:** Anne Leppänen, Peter Lindgren, Carl Johan Sundberg, Max Petzold, Tanja Tomson

**Affiliations:** 1 Department of Learning, Informatics, Management and Ethics, Karolinska Institutet, Stockholm, Sweden; 2 The Swedish Institute for Health Economics, Lund, Sweden; 3 Department of Physiology and Pharmacology, Karolinska Institutet, Stockholm, Sweden; 4 School of Public Health and Community Medicine, Institute of Medicine, University of Gothenburg, Gothenburg, Sweden; University of New Mexico, UNITED STATES

## Abstract

**Objective:**

To evaluate the effectiveness of Tobacco Cessation on Prescription (TCP) compared to standard treatment in socioeconomically disadvantaged areas in Swedish primary healthcare (PHC).

**Study design:**

A pragmatic cluster randomized controlled trial, where randomization was conducted at the PHC center level using a computer-generated random allocation sequence.

**Setting:**

18 PHC centers in socioeconomically disadvantaged areas in Stockholm.

**Participants:**

250 adult daily tobacco users (56% female, 41% foreign born) with Swedish social security numbers and permanent resident permits, fluent in Swedish or Arabic, of which 140 responded to the follow-up at 6 months and 139 to the follow-up at 12 months. No blinding was applied.

**Interventions:**

TCP (tobacco cessation counseling for ≥10 minutes, an individualized prescription for tobacco cessation treatment and follow-up on ≥1 occasion) compared to standard treatment.

**Primary and secondary outcome measures:**

The primary outcome was self-reported 7-day abstinence at 6 months and the secondary outcomes included self-reported 7-day abstinence at 12 months and 3-month continued abstinence at 6 and 12 months follow-up.

**Results:**

PHC centers were randomized to the intervention group (n = 8) and control group (n = 10). At the PHC centers, 250 patients (TCP n = 188, standard treatment n = 62) were recruited. There was a statistically significant effect of TCP compared to standard treatment for the outcomes 7-day abstinence at 6 months (OR adjusted 5.4, 95% CI 1.57 to 18.93) and 3-month continued abstinence at 6 (OR adjusted 6.4, 95% CI 1.30 to 31.27) and 12 months follow-up (OR adjusted 7.8, 95% CI 1.25 to 48.82).

**Conclusions:**

TCP may be effective in achieving abstinence from tobacco use compared to standard treatment in the given setting but due to several limitations, resulting in high attrition rates and a low statistical power in the study, more research is needed to evaluate this.

**Trial registration:**

ISRCTN 11498135.

## Introduction

Tobacco use is responsible for approximately 8% of the total disease burden in Sweden [1]. Although the prevalence of daily smoking in Sweden has decreased to 6% in the general population, it is more than four times higher among socioeconomically disadvantaged groups compared to their counterparts [2]. In addition, the smokeless tobacco product snus is used on a daily basis by 20% of men and 6% of women [2].

To tackle the harmful effects of tobacco use and related inequalities in health, clinical guidelines for tobacco cessation have been developed [3, 4]. They recommend provision of qualified tobacco cessation counseling, combined with nicotine replacement therapy or prescription drugs (varenicline and bupropion), to support daily smokers to quit [4]. They also state that support to high-risk groups such as lower socioeconomic groups should be given priority [4].

Primary healthcare (PHC) has an important role in providing tobacco cessation treatment since it has the main responsibility for health promotion in the Swedish healthcare system [5] and the majority of the population has regular contact with PHC. Individuals from lower socioeconomic groups also visit PHC more often than those from higher socioeconomic groups [6]. However, socioeconomically disadvantaged groups appear to face specific challenges in quitting their tobacco use [7–9] and PHC providers in Sweden and elsewhere have reported several barriers to work with tobacco cessation [9–11].

In Sweden and in many other countries, prescription approaches are used in PHC to promote physical activity among patients [12]. A similar intervention has been developed by the authors to promote tobacco cessation in Swedish PHC. It is called Tobacco Cessation on Prescription (TCP) and consists of tobacco cessation counseling for ≥10 minutes, a written prescription for individualized tobacco cessation treatment and follow-up on ≥1 occasion [13].

In previous qualitative studies we have explored patients’ and PHC providers’ experiences of TCP [9, 14, 15], showing that it was perceived as a useful tool that could facilitate a more structured and effective approach to tobacco cessation compared to current practices targeting socioeconomically disadvantaged groups in PHC [14]. Findings also suggest that TCP may have an impact on patient and PHC provider behavior, leading to decreased tobacco use among patients [9, 15] but this has previously not been evaluated. Therefore, the aim of this study was to evaluate the effectiveness of TCP compared to standard treatment for tobacco cessation in socioeconomically disadvantaged areas in Swedish PHC.

## Methods

### Study design

A two-armed pragmatic cluster randomized controlled trial was chosen as the study design. Randomization was conducted at the PHC center level, meaning that participating PHC centers were randomly allocated to intervention or control conditions. At the PHC centers, all patients in the intervention arm were offered TCP and all patients in the control arm were offered standard treatment. Patient characteristics were measured at baseline and outcomes at 6 and 12 months after the intervention. The trial has been approved by the Regional Ethical Review Board in Stockholm [ref: 2015/207-31, 2015/1226-32, 2016/2080-32]. It is presented according to the CONSORT Statement with relevant extensions [16–18] to ensure quality in the reporting (summary shown in S1 File. CONSORT Checklist). The original study protocol submitted to the Ethical Review Board in Stockholm is provided in Swedish in S2 File and translated to English in S3 File. An updated version with further details is reported in the published study protocol [13]. The authors confirm that all ongoing and related trials for this intervention are registered.

### Study setting and participants

Eligible PHC centers included PHC centers in socioeconomically disadvantaged areas in Stockholm. Eligible patients included daily tobacco users over 18 years of age with Swedish social security numbers and permanent residence permits, fluent in Swedish or Arabic with willingness to participate in the study. Daily tobacco use was defined as use of cigarettes, snus or other tobacco products every day for the past year. Ongoing treatment for tobacco cessation and cognitive impairment affecting voluntariness to participate were applied as exclusion criteria.

### Sampling and recruitment

PHC centers were sampled based on a socioeconomic index considering the income, educational level, ethnicity and health status of the population in a PHC center’s catchment area [19]. Managers at the PHC centers were contacted via telephone by the researchers and offered further information via e-mail and a physical meeting before agreeing to participate. PHC centers were offered reimbursement (corresponding to 20% of a nurse’s monthly salary for three months and 10% for another three months plus 100 SEK per recruited patient) to cover the administrative costs of participating in the study.

Patients were recruited by one to three appointed staff members employed at each of the participating PHC centers. Most appointed staff members were nurses, specialized in diabetes or lung diseases. Other staff members could also refer patients to the responsible employees. Eligible patients were identified through a short screening questionnaire and invited to participate. Further information about the study was administered by responsible staff at the participating PHC centers and written informed consent was sought from all participants. Patients were offered a 100 SEK gift certificate as an incentive to participate.

### Treatments

#### Tobacco Cessation on Prescription (intervention)

TCP included 1) tobacco cessation counseling for ≥10 minutes, 2) a written prescription for individualized tobacco cessation treatment and 3) follow-up on ≥1 occasion. The TCP prescription form included options for further counseling (individual, group or telephone-based counseling in PHC, dental care or at the Swedish National Tobacco Quitline), pharmacotherapy (nicotine replacement therapy, varenicline, bupropion), other measures for tobacco cessation (physical activity and other strategies to cope with withdrawal symptoms), follow-up (by telephone or revisit) and support for self-management (questions for self-reflection and reference to mobile applications and websites). The prescription form was available on paper and electronically as a PDF-file. The form could be used as a basis for, or summary, of the counseling or as a written agreement. It was recommended that the patients in the study were given a copy of the filled out prescription form but the PHC providers were free to use the prescription form as they preferred. PHC providers responsible for the treatment of patients in the intervention group received 3.5 hours of training in tobacco cessation and TCP. A manual summarizing the training was also distributed.

#### Standard treatment (control)

Standard treatment was defined as treatment for tobacco cessation according to current practice at the PHC centers. Responsible staff members were free to offer whatever cessation treatment they wanted as long as it was documented in a study-specific protocol for later comparison with TCP. The minimum treatment for the control group was 5 minutes of counseling. The control group did not have access to the TCP prescription form so the main difference between the treatment arms was how the counseling was administered (with or without a prescription form). PHC providers responsible for the treatment of patients in the control group received the same training as providers in the intervention group, excluding 30 minutes of information about TCP. A manual identical to the one developed for the intervention group but excluding all information about TCP, was also distributed to providers in the control group.

### Outcomes

As recommended by experts, the primary outcome was measured in self-reported point prevalence of 7-day abstinence (total abstinence from tobacco use during the 7 days preceding follow-up) at 6 months follow-up [20]. The secondary outcomes were measured in self-reported 7-day abstinence at 12 months, as well as 3-month continued abstinence (total abstinence from tobacco during the 90 days preceding follow-up), any cigarette quit attempt (total abstinence from cigarettes >24 hours) among daily smokers, daily tobacco use (number of cigarettes per day during the last 7 days) among non-quitters and change in health-related quality of life (based on a weighted index value from 0 to 1 where 0 represents death and 1 represents perfect health) at 6 and 12 months follow-up.

The following changes to outcomes were made to what was stated in the published study protocol. Quit attempts and daily tobacco use were only reported for cigarettes since 97% of the study participants were daily smokers and snus and cigarettes were not considered appropriate to merge in the analysis of these outcomes. Daily cigarette use was only reported for non-quitters to evaluate the effect of reduced use separately from the effect of cessation. Since there was a difference in health-related quality of life between the treatment groups at baseline, this outcome was reported as change instead of absolute numbers at follow-up.

### Data collection

Data on sociodemographic characteristics, tobacco use, cessation, health status and health-related quality of life was collected through paper-based questionnaires. The questions were based on the Swedish Public Health Survey 2014 [21], a questionnaire used to evaluate the effectiveness of brief advice for tobacco cessation in Swedish dental clinics [22] and the Swedish and Arabic (Lebanon) version of EQ-5D-5L [23]. The questionnaires were pilot tested in Swedish prior to the start of the study. They were also translated to Arabic and pilot tested prior to the recruitment of Arabic speaking participants.

The measurements were conducted at baseline and at 6 and 12 months after the intervention. In the follow-up questionnaires, questions about tobacco cessation support received were added. Responsible staff administered the baseline questionnaires and documented the cessation treatment in the electronic medical record as usual and in study-specific documentation protocols to enable a structured comparison of the interventions. They received written information and training in the documentation procedures before their involvement in the study. The researchers visited the PHC centers every three to four months to collect data and consent forms and to ensure that the study procedures were followed or adapted if needed, *e*.*g*. if new or additional PHC providers needed training to participate in the study. The latter occurred at approximately half of the PHC centers.

The 6- and 12 month follow-up questionnaires were sent to all participants via mail, regardless of their response to the 6-month follow-up. If the follow-up questionnaires were not returned within ten days, a reminder with a new questionnaire was sent via mail. If this was not returned within ten days, additional reminders were sent via mail, e-mail, and Short Message Service (SMS) with the option to answer the questionnaire in a telephone interview. All follow-up questionnaires and reminders were sent by the researchers.

Data on the characteristics of participating PHC centers were collected by the researchers through informal interviews with staff members at baseline, including data on number of listed patients and employees at the PHC centers.

### Sample size

The sample size was calculated by the researchers based on the primary outcome, assuming a 7% point prevalence of 7-day abstinence at 6 months follow-up in the control group, a significance level of 5% and 80% power to detect a relative risk of 2.0 when comparing the treatment groups, leading to an unadjusted sample size of 300 participants per arm. Assuming an intra-cluster coefficient (ICC) of 0.01 [24] the sample size was adjusted for design effect using the formula: 1 + (m − 1) * ICC, where m represented the mean number of participants in each cluster. In the sample size calculation, 7 clusters per arm and 43 participants per cluster were assumed, leading to a sample size of 426 participants per arm. Adjusted to an estimated attrition rate of 8%, the final sample size required was 464 participants per arm or 928 in total.

### Randomization

Due to recruitment challenges, the PHC centers were randomized in two sets. In the first set, PHC centers were paired based on their socioeconomic index and allocation to treatment/control was done per pair using the uniform random number generator built into Excel. Allocation was done with a 1:1 ratio. In the second set, the randomization was not paired based on socioeconomic index, the ratio 4:6 between the intervention and control group was used due to a higher number of PHC centers dropping out in the control arm. The randomizations were conducted and communicated to the PHC centers by the researchers. Cluster randomization was employed at the PHC center level to avoid contamination of the trial conditions. This meant that all patients recruited from a particular PHC center were allocated to receive the same treatment.

### Blinding

No blinding was applied. However, patients were only informed that they would be randomly allocated to receive different types of tobacco cessation support when they were recruited to participate. Thus, they were not informed about their treatment allocation or what the trial conditions entailed until after the study. This was done to avoid attrition and preconceptions regarding the effectiveness of treatment [25]. PHC providers could not be blinded since they were responsible for the treatment of patients and the researchers could not be blinded due to constant interaction with the participating PHC centers when training staff members and manually collecting and managing collected data.

### Statistical analysis

Descriptive statistics of baseline characteristics at the individual and cluster level are presented separately for the treatment groups. Categorical variables are reported as proportions and continuous variables as mean values with standard deviations (SDs). All descriptive statistics were analyzed in IBM SPSS Statistics 25.

Associations between treatment and outcomes at 6 and 12 months follow-up were analyzed in multiple regression models. Logistic regression models were used for binary outcomes, including point prevalence of 7-day abstinence. The results are presented as odds ratios (ORs) with 95% confidence intervals (CIs). Associations between treatment and continuous/count outcomes, including cigarettes per day among non-quitters and change in health-related quality of life, were analyzed in multiple linear and Poisson regression models. All analyses were conducted according to the intention-to-treat principle [26], meaning that individual participants were analyzed according to how the PHC centers they were recruited at were randomized, regardless of the treatment they received. Inference was targeted at the individual level and hierarchical models were used to handle clustering on the PHC center level. Model covariates at the patient level were pre-defined in the study protocol and included age, gender, educational level, chronic disease diagnosis, nicotine dependence, previous quit attempts, ever-use of pharmacotherapy and importance and intention to quit [13]. All baseline characteristics on the PHC center level were also included. Due to a smaller sample size than expected the models were reduced by removing all non-significant covariates in the final models. This to avoid non-convergence in the iterative estimation process of the hierarchical models. As a sensitivity analysis, the results from the hierarchical models were confirmed in non-hierarchical models for the primary analysis without altering significances or intervention effect directions in any of the outcomes. The goodness-of-fit was assessed using Hosmer-Lemeshow test for logistic regression and by assessing relation between residuals and covariates in the linear regressions. The observed deviations from expected levels were judged to be minor.

Before data was analyzed, logical assumptions were made to handle drop-out in responses to individual questionnaire items. For example, multiple answers to highest education achieved was interpreted as the highest education reported. Where logical assumptions could not be made (*e*.*g*. no response to intention to quit), data was defined as missing. No sensitivity analysis with imputed values was needed since none of the variables in the models had more than 5% missing values, as specified in the study protocol [13]. However, a sensitivity analysis of the primary outcome was conducted assuming that all non-respondents at 6- month follow-up had continued their tobacco use. Exploratory analysis of cessation treatment and perceptions of the support received was also conducted. All regression models were analyzed in Stata 15.1.

## Results

**Fig 1:**
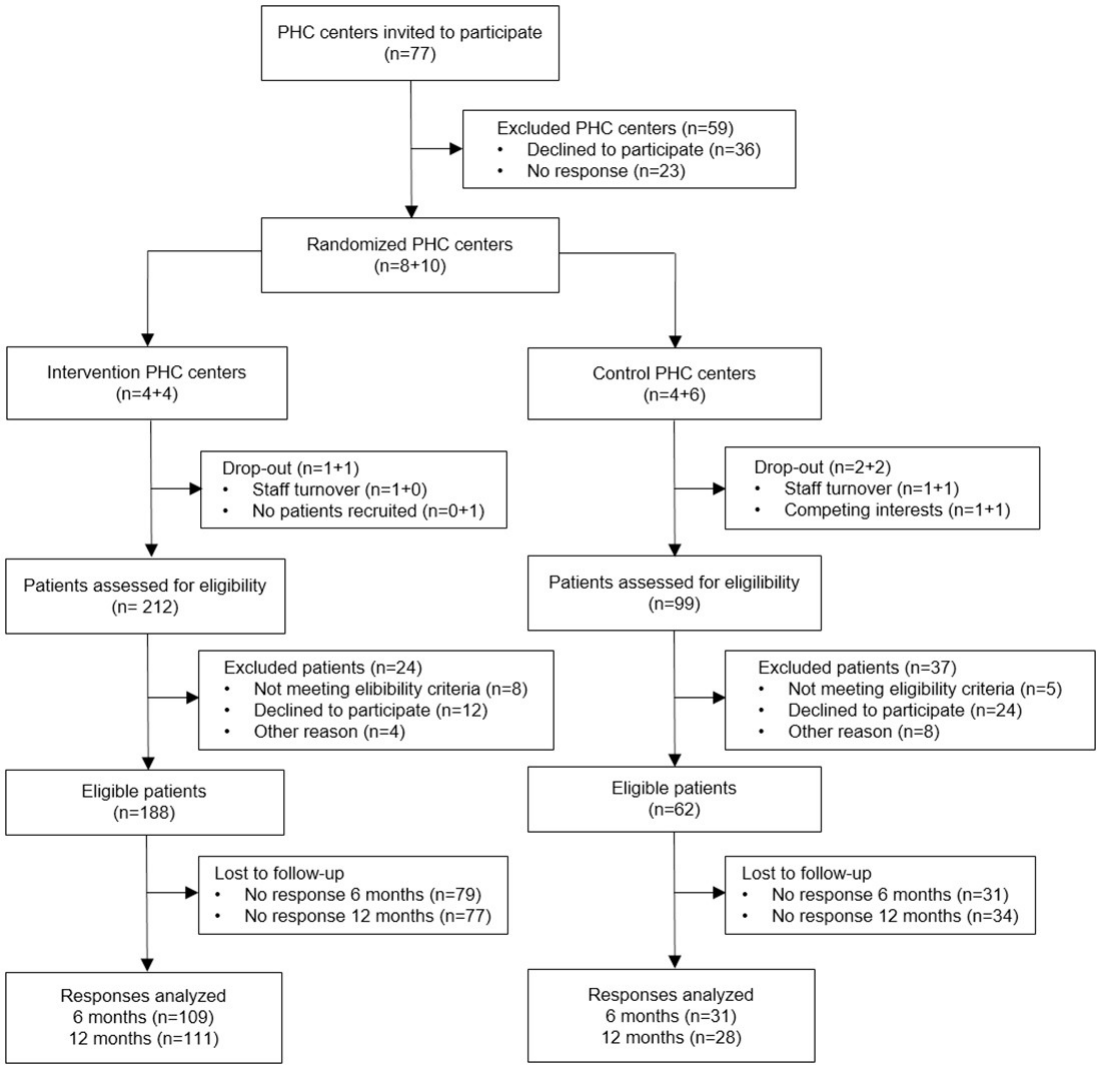
CONSORT flow diagram.

From April 2015 to August 2016, 77 PHC centers were invited and 18 agreed to participate. Reported reasons for non-participation were high workload, staff turnover and lack of resources. Excluded PHC centers had a lower socioeconomic index (more affluent) and were more often privately operated compared to included PHC centers. After randomization was completed, six PHC centers dropped out of the study due to staff turnover and organizational changes. These PHC centers had a higher socioeconomic index (more socioeconomically disadvantaged) and were more often publicly operated compared to those who continued in the study. Patients were recruited from February 2016 to August 2018. Patient recruitment took markedly longer than expected and had to be interrupted before the estimated sample size could be obtained. In total, 311 patients were screened for eligibility and 250 were included in the study. Patients who did not meet the eligibility criteria, who declined to participate or were missing signed consent forms and/or baseline data were excluded from the study. Excluded patients were of similar age as included patients but more often male. The majority of patients screened (68%) were in the intervention group. The participation rate was also higher in the intervention group compared to the control group (89% and 63% respectively). In addition, most of the participants (75%) were recruited from the PHC centers in the intervention group. Patients were followed-up from August 2016 to September 2019. In total, 140 patients (56%) responded to the follow-up at 6 months and 139 patients (56%) responded to the follow-up at 12 months. The response rate was higher in the intervention group compared to the control group (58% compared to 50% at 6 months and 59% compared to 45% at 12 months follow-up). Patients lost to follow-up were somewhat younger, more often male, employed and foreign born compared to respondents. Further details on the PHC center and participant flow are presented in Fig 1.

PHC centers in the intervention group had fewer patients listed, more employees, lower socioeconomic index (more affluent) and were more often privately operated compared to PHC centers in the control group. For further details, see Table 1.

**Table 1:** PHC center characteristics and patient demographics at baseline.

	Control	Intervention	Total
PHC centers	n = 10	n = 8	n = 18
Socioeconomic index			
Mean (SD)	0.431 (0.042)	0.422 (0.055)	0.427 (0.047)
Number of patients listed^a^			
Mean (SD)	9 150 (4 529)	8 781 (8 472)	8 966 (6 565)
Number of employees^a^			
Mean (SD)	23.2 (7.7)	30.3 (29.3)	26.8 (21.0)
Operation, n (%)			
Public	7 (70)	3 (37.5)	10 (56)
Private	3 (30)	5 (62.5)	8 (44)
Patients	n = 62 (%)	n = 188 (%)	n = 250 (%)
Questionnaire language			
Swedish	59 (95)	177 (94)	236 (94)
Arabic	3 (5)	11 (6)	14 (6)
Gender			
Female	31 (50)	110 (58.5)	141 (56)
Male	31 (50)	78 (41.5)	109 (44)
Age			
18–39	13 (21)	28 (15)	41 (16)
40–49	8 (13)	35 (19)	43 (17)
50–59	16 (26)	51 (27)	67 (27)
>60	25 (40)	74 (39)	99 (40)
Mean (SD)	54.0 (14.6)	54.5 (14.5)	54.4 (14.5)
Education			
Elementary	17 (27)	46 (25)	63 (25)
Secondary	25 (40)	76 (40)	101 (40.5)
Post-secondary	14 (23)	41 (22)	55 (22)
Other	6 (10)	25 (13)	31 (12.5)
Occupation			
Employed	29 (47)	89 (47)	118 (47)
Not employed	33 (53)	99 (53)	132 (53)
Foreign born			
Yes	33 (53)	69 (37)	102 (41)
No	29 (47)	119 (63)	148 (59)
Any chronic disease			
Yes	47 (76)	129 (69)	176 (70)
No	15 (24)	59 (31)	74 (30)
EQ-5D-5L index			
Mean (SD)	0.71 (0.27)	0.74 (0.22)	0.73 (0.23)

^a^ Data from two PHC centers in the control group missing

Patients generally had similar characteristics in the treatment groups but patients in the intervention group were more often female, Swedish born, had more previous quit attempts, previous experience of pharmacotherapy and lower prevalence of chronic disease compared to the control group. Patient demographics at baseline are presented in Table 1 and further details on patients’ tobacco use at baseline are presented in Table 2.

**Table 2:** Patients’ tobacco use at baseline.

	Control	Intervention	Total
	n = 62 (%)	n = 188 (%)	n = 250 (%)
**Type of tobacco** ^a^			
Cigarettes	60 (97)	186 (99)	246 (98)
Snus	5 (8)	11 (6)	16 (6)
Other	1 (2)	3 (2)	4 (2)
**Daily tobacco use (number of cigarettes)** ^b^			
≤10	19 (32)	55 (29.5)	74 (30)
11–20	36 (60)	107 (57.5)	143 (58)
>20	5 (8)	24 (13)	29 (12)
Mean (SD)	15.1 (7.4)	15.7 (7.8)	15.6 (7.7)
**Duration of tobacco use (years)**			
≤10	6 (9.5)	17 (9)	23 (9)
11–20	9 (14.5)	28 (15)	37 (15)
>20	47 (76)	140 (76)	187 (76)
Mean (SD)	32.6 (15.4)	34.0 (14.7)	33.6 (14.9)
**Time to first use in the morning (minutes)**			
≤5	19 (31)	46 (25)	65 (26)
6–30	29 (48)	89 (48)	118 (48)
31–60	8 (13)	33 (18)	41 (17)
>60	5 (8)	17 (9)	22 (9)
**Heaviness of Smoking Index (nicotine dependence)** ^**b**^			
Low	7 (12)	37 (20)	44 (18)
Moderate	34 (57.5)	100 (55)	134 (55.5)
High	18 (30.5)	46 (25)	64 (26.5)
**Previous cigarette quit attempts**			
0	19 (32)	36 (20)	55 (23)
1	8 (13)	20 (11)	28 (12)
2–3	7 (11.5)	31 (17)	38 (16)
4–5	7 (11.5)	27 (15)	34 (14)
≥6	19 (32)	66 (37)	85 (35)
**Ever-use of pharmacotherapy**			
Yes	40 (64.5)	134 (71)	174 (70)
No	22 (35.5)	54 (29)	76 (30)
**Importance to quit**			
Low	2 (3)	2 (1)	4 (2)
Moderate	7 (11)	19 (10)	26 (10)
High	53 (86)	167 (89)	220 (88)
**Self-efficacy to quit**			
Low	14 (23)	25 (13)	39 (16)
Moderate	20 (32)	88 (47)	108 (43)
High	28 (45)	75 (40)	103 (41)
**Intention to quit**			
Within 1 month	32 (52)	88 (48)	120 (49)
Within 2–6 months	16 (26)	65 (35.5)	81 (33)
>6 months	7 (11)	10 (5.5)	17 (7)
Time undefined	7 (11)	20 (11)	27 (11)

^a^Patients could be daily users of more than one type of tobacco product

^b^Only including daily cigarette smokers

Almost all patients responded to the follow-up in Swedish (94%) and by paper-based questionnaire (94%) as opposed to in a telephone interview. In total, 38 of 108 patients in the intervention group and 4 of 31 patients in the control group reported total abstinence from tobacco in the 7 days preceding follow-up at 6 months after the intervention. When adjusted for significant covariates, the odds ratio for the primary outcome was 5.4. The adjusted odds ratio for 3-month continued abstinence was 6.4 at 6 months and 7.8 at 12 months follow-up. These associations were statistically significant. There were no statistically significant differences between the treatment groups in the other outcomes. Further details on all outcomes are presented in Table 3. Full model results are presented in S4 File. For binary outcomes, logit results and corresponding 95% CIs are presented in S5 File.

**Table 3:** Treatment outcomes at 6 and 12 months follow-up.

	6 months				12 months			
	Control	Intervention			Control	Intervention		
	Outcome/number analyzed (%)		OR (95% CI) unadjusted	OR (95% CI) adjusted	Outcome/number analyzed (%)		OR (95% CI) unadjusted	OR (95% CI) adjusted^a^
**7-day abstinence**	4/31 (13)	38/108 (35)	**3.5 (1.06–11.90)**	**5.4 (1.57–18.93)** ^**a**^	4/28 (14)	41/111 (37)	**3.4 (1.03–11.15)**	3.4 (0.98–11.45)^a^
**3-month abstinence**	3/31 (10)	29/109 (27)	3.4 (0.96–11.98)	**6.4 (1.30–31.27)** ^b^	2/27 (7)	34/108 (31)	**5.7 (1.27–25.81)**	**7.8 (1.25–48.82)** ^f^
**Any cigarette quit attempt**	11/22 (50)	50/85 (59)	1.5 (0.46–4.85)	1.1 (0.40–3.23)^c^	11/24 (46)	35/76 (46)	1.0 (0.40–2.53)	0.7 (0.25–1.92)^g^
	**Mean (SD)**		**p-value unadjusted**	**p-value adjusted**	**Mean (SD)**		**p-value unadjusted**	**p-value adjusted**
**Cigarettes per day among non-quitters**	11.4 (7.4)	11.9 (7.8)	0.754	0.754^d^	10.6 (8.7)	10.9 (6.0)	0.879	0.757^h^
**Change in EQ-5D-index**	0.01 (0.19)	-0.02 (0.17)	0.547	0.919^e^	0.01 (0.25)	0.00 (0.23)	0.781	0.994^i^

Adjusted for covariates with a significance level of <0.05

^a^ gender, intention to quit and PHC operation

^b^ gender, intention to quit, PHC operation, number of listed patients and employees

^c^ previous cigarette quit attempts

^d^ no significant covariates

^e^ intention to quit and importance to quit

^a^ gender, intention to quit and PHC operation

^f^ gender, intention to quit, PHC operation and socioeconomic index

^g^ importance to quit

^h^ Heaviness of Smoking Index and ever-use pharmacotherapy

^i^ importance to quit

Sensitivity analysis was conducted on the primary outcome, assuming that all non-respondents had continued their tobacco use at 6 months follow-up. The sensitivity analysis produced lower odds ratios (OR unadjusted 3.6, 95% CI 1.20 to 10.95, OR adjusted for gender 3.5, 95% CI 1.18 to 10.30) with statistically significant associations.

Exploratory analysis of the cessation treatment provided showed that the mean (SD) number of counseling sessions was 2.6 (1.8) in the intervention group and 2.4 (1.5) in the control group (p = 0.924). Furthermore, the mean (SD) number of counseling minutes was 105 (56) in the intervention group compared to 89 (51) in the control group (p = 0.889). These differences were not statistically significant. The content of the counseling was also similar in the treatment groups but there was a statistically significant difference in the proportion of patients in the intervention group that received counseling on pharmacotherapy compared to the control group (OR 5.2, 95% CI 1.29 to 21.06). A higher proportion of patients in the intervention group also used prescription drugs for tobacco cessation (varenicline or bupropion) (OR 2.9, 95% CI 0.82 to 10.28) and considered the support they had received as sufficient (OR 1.5, 95% CI 0.33 to 7.36) at 6 months follow-up. However, these associations were not statistically significant, nor was the difference in use of nicotine replacement therapy at 6 months follow-up (OR 0.8, 95% CI 0.36 to 2.00).

## Discussion

To the best of the authors’ knowledge, this is the first study to evaluate the effectiveness of TCP in Swedish PHC. When adjusted for significant covariates at baseline, the odds of achieving 7-day abstinence was 5.4 times higher among patients receiving TCP compared to standard treatment at 6 months follow-up. The adjusted odds ratio for 3-month continued abstinence was 6.4 at 6 months follow-up and 7.8 at 12 months follow-up. These associations were statistically significant. However, the sensitivity analysis of the primary outcome yielded a lower odds ratio, indicating that 7-day abstinence at 6 months may have been overestimated. A previous systematic review in the field reported relative risks around 1.2 to 2.5 for effective tobacco cessation treatments [27], while the unadjusted relative risk recalculated for the primary outcome in Table 3 was 2.7, suggesting that the effect size is relatively high and can be considered clinically relevant. However, there are several limitations in the study that should be considered when interpreting the results.

The attrition rate was relatively high, particularly among more disadvantaged participants. It was higher in this study compared to other tobacco cessation studies in Sweden [22, 28] but attrition rates between 10.8 and 77% have been reported elsewhere, with the highest rates found among more disadvantaged groups [29]. Personalized telephone interviews or web-based questionnaires could have increased the response rate in this target group [30].

Difficulties with recruiting and retaining PHC centers and patients could also be explained by high staff turnover, workload, and organizational changes in PHC as these were the main barriers for PHC centers to engage in the study. PHC centers with one staff member responsible for tobacco cessation were particularly vulnerable to this. Lone responsibility to work with tobacco cessation has previously been emphasized as a barrier to continuity, negatively affecting access to cessation treatment [9, 15]. It also resulted in a smaller sample size and higher level of uncertainty in the results than expected. This is reflected in the wide confidence intervals for the odds ratios reported in the results. In addition, large differences in cluster sizes (1–29 observations per cluster at follow-up) may have introduced uncertainty in the cluster analysis. The results from the primary analysis were confirmed in non-hierarchical models without any material effects on the outcomes, indicating some level of robustness. In the sample size calculation, an ICC of 0.01 was assumed. For most of the outcomes the ICC was 0.01 or below. For the unadjusted analysis of the outcomes 7-day abstinence and any cigarette quit attempt at 6 months, the ICCs were 0.028 and 0.069, respectively, which decreased the power in these analyzes. Similar challenges were found for the unadjusted analysis of the outcomes 7-day abstinence and change in EQ-5D index at 12 months. Randomization at the individual level could have increased the statistical power in the study since this would have required fewer participants than the corresponding cluster randomized trial [31]. However, cluster randomization was considered necessary as few staff members at the PHC centers had the ability to provide cessation support and randomization at the patient level could have introduced contamination of the trial conditions.

Another limitation in the study was that the PHC providers were aware of the treatment allocation. This may have had a negative effect on PHC providers’ motivation to recruit patients and offer cessation support in the control arm, potentially contributing to lower recruitment and abstinence rates among patients in this arm. Still, individual study participants were not informed about the treatment options in the study arms until after the study, reducing the likelihood of biased psychological or physical responses to treatment among patients [25].

An additional limitation of the study was the reduction of the statistical models to enable convergence in the iterative estimation procedures. Insignificant covariates were removed from the models which could be argued to be data driven and decreasing the inference from the models. This limitation in evidence has been acknowledge by careful presentation, interpretation, and discussion of the results.

The effect sizes in this study were larger than expected and yielded statistically significant and clinically relevant treatment effects for several outcomes. A possible explanation for this may be the low statistical power in the study. Another explanation could be increased self-efficacy among PHC providers to work with tobacco cessation and increased involvement in the treatment among patients with TCP, as reported in a previous study exploring PHC providers’ perceived barriers and facilitators of implementing TCP [9]. However, these and other emotional aspects of the treatment were not captured in this study. The exploratory analysis showed that a higher proportion of patients were given advice about pharmacotherapy for tobacco cessation in the intervention group compared to the control group but no other statistically significant differences between the treatment groups were found in the amount or content of the counseling. Thus, more research is needed to explore the potential mechanisms of TCP.

A strength of the study was the pragmatic approach applied, reflecting real world conditions, and providing high external validity of the findings [32]. This may have increased the generalizability of the results to similar settings, for example to other regions in Sweden. However, a relatively large number of PHC centers and patients that were invited declined to participate, increasing the risk of selection bias. Reported reasons for non-participation among the PHC centers were related to organizational challenges, indicating that the participating PHC centers may have had better prerequisites to work with tobacco cessation. Furthermore, most of the patients that participated reported a high motivation and intention to quit their tobacco use, suggesting that they may have been more motivated to change behavior and receive treatment compared to those who declined to participate in the study. The small sample size, high attrition rate and difference between drop-outs and respondents may have reduced the generalizability of the findings [29]. Given the methodological challenges in the study, more research is needed to evaluate the effectiveness of TCP. Future studies should employ strategies to further strengthen recruitment and retention of PHC centers and patients, for example by providing the option to respond to questionnaires, engage in cessation treatment, research administration and training online, and having at least two PHC providers responsible for cessation treatment at each PHC center.

## Conclusions

The results suggest that TCP may be effective in achieving abstinence from tobacco use compared to standard treatment in socioeconomically disadvantaged areas in Swedish PHC but due to several limitations, resulting in high attrition rates and a low statistical power in the study, more research is needed to evaluate this.

## Supporting information

S1 File CONSORT checklist. (DOCX)

S2 File Original study protocol in Swedish. (PDF)

S3 File Translated study protocol in English. (PDF)

S4 File Full model results. (DOCX)

S5 File Logit results for binary outcomes. (DOCX)
