# Supplementary material for: Motivation 2 Quit (M2Q): A cluster randomized controlled trial evaluating the effectiveness of Tobacco Cessation on Prescription in Swedish primary healthcare
Source: PLoS One. 2022 Dec 1;17(12):e0278369. doi: 10.1371/journal.pone.0278369 (PMC9714827; doi:10.1371/journal.pone.0278369)
Supplement: S2 File — (PDF) [file pone.0278369.s002.pdf]

Bilaga 2

Forskningsplan för Tobaksavvänjning på recept som en primärvårdsintervention i utsatta områden i Stockholms län

Vetenskapliga frågeställningar

Det övergripande syftet med projektet är att undersöka om en receptmetod kan användas för att facilitera tobaksavvänjning bland socioekonomiskt utsatta grupper i svensk primärvård. Detta kommer att uppnås genom att besvara följande frågeställningar:

1. Vad är effektiviteten av ToR jämfört med nuvarande strategier för tobaksavvänjning i svensk primärvård? (Studie I)

2. Vad är kostnadseffektiviteten av ToR jämfört med nuvarande strategier för tobaksavvänjning i svensk primärvård? (Studie II)

3. Vilka är de upplevda hindrande och möjliggörande faktorerna för implementeringen av ToR som en primärvårdsintervention i socioekonomiskt utsatta områden i Sverige? (Studie III)

Områdesöversikt

Tobaksbruk ses som den ledande förebyggbara dödsorsaken i världen (1) och beräknas orsaka cirka en tiondel av alla dödsfall i Sverige (2). Dessutom är kronisk sjukdom till följd av tobaksbruk ett stort folkhälsoproblem i Sverige – framförallt i socioekonomiskt utsatta grupper där förekomsten av tobaksbruk är mycket högre än i den generella befolkningen (3). Utöver den negativa inverkan tobak har på hälsan och livskvaliteten hos befolkningen (4) är den även förenad med ökade kostnader för hälso- och sjukvården men även för samhället i stort (5). Tobaksavvänjning har visat sig minska risken för att i förtid insjukna och dö i tobaksrelaterade sjukdomar (6), varför detta är ett prioriterat målområde i den svenska folkhälsopolitiken (7). I Sverige har Socialstyrelsen utfärdat Nationella riktlinjer för sjukdomsförebyggande metoder, där åtgärder för tobaksavvänjning ingår (8). Dessa inkluderar insatser som enkla råd, rådgivande samtal (i kombination med nikotinläkemedel eller särskild övervakning), kvalificerat rådgivande samtal (i kombination med nikotinläkemedel, vareniklin eller bupropion), proaktiv telefonrådgivning samt webb- och datorbaserad rådgivning (8). Riktlinjerna rekommenderar att vårdgivare bör erbjuda alla tobaksanvändare stöd till avvänjning (8). Trots detta är behandlingsintensiteten för tobaksavvänjning relativt låg (9)och man misslyckas ofta med att nå högriskgrupper som har ett större behov av att få hjälp (8). Svårigheterna kan bero på ett lägre socialt stöd, lägre motivation till avvänjning, starkare tobaksmissbruk, ökad sannolikhet att avbryta läkemedelsbehandling och stöd för beteendeförändring, samt psykologiska skillnader som ökad mottaglighet för marknadsföring från tobaksindustrin (10). Behovet av ett mer systematiskt arbete med tobaksavvänjning i sjukvården och en bättre tillgång till avvänjningsstöd för socioekonomiskt utsatta grupper lyftes nyligen (11).

Bilaga 2

Studier gjorda i Stockholm på vårdkonsumtion i olika sociala grupper visar att individer av utländskt ursprung, låg utbildningsnivå samt lägre inkomst ofta använder sig av primärvården (12). Allmänheten har stort förtroende för sjukvården och de flesta som använder tobak söker vård för olika hälsoproblem på vårdcentraler. Primärvården har därmed en betydande potential som stöd vid tobaksavvänjning.

I en förstudie utreddes nyligen den upplevda acceptansen, genomförbarheten samt för- och nackdelarna av att använda Tobaksavvänjning på recept (ToR) som ett verktyg för att nå ut med hälsofrämjande insatser till socioekonomiskt utsatta grupper inom primärvården i Stockholm (13). Resultaten baserades på semistrukturerade intervjuer med klienter, vårdpersonal och experter inom andra livsstilsinsatser på recept (13). Utöver önskemål på innehåll och utformning av ToR, fann studien att verktyget skulle ha en positiv emotionell betydelse för klienter (t.ex. vad gäller motivation och upplevd "rätt" att söka vård för tobaksavvänjning) medan den skulle ha en positiv praktisk betydelse för vårdpersonal (underlättande vid planering, dokumentation, etc.) (13).

Förhoppningen är att ToR i framtiden ska kunna implementeras och förskrivas på ett liknande sätt som Fysisk aktivitet på recept (FaR), en annan livsstilsinsats på recept som visat sig förbättra hälsa och livskvalitet och som redan används för att förebygga kronisk sjukdom i Sverige (14). Förskrivning av livsstilsinsatser på recept kan leda till att patienter tar råd på större allvar då de ges av en person med en läkares/sjukvårdspersonals auktoritet. Dessutom kan benägenheten att förändra beteenden öka om tydliga råd ges och ordineras med samma engagemang och övertygelse som läkemedel (15). ToR kan därmed ses som ett möjligt verktyg för att underlätta och systematisera primärvårdens arbete med tobaksprevention enligt befintliga riktlinjer (13). Trots förväntade fördelar har ett recept för tobaksavvänjning tidigare inte utvärderats.

Projektbeskrivning

Projektet kommer att bestå av tre delstudier som ämnas genomföras under 2015-2019 som en del av ett doktorandprojekt vid Institutionen för Lärande, Informatik, Management och Etik vid Karolinska Institutet. Projektet finansieras av Stockholms Läns Landsting.

Studie I

Cirka 654 studiedeltagare kommer att rekryteras från 14 vårdcentraler i socioekonomiskt utsatta områden i Stockholm. Lämpliga vårdcentraler kommer att identifieras av forskargruppen baserat på ett socioekonomiskt index (16) och ändamålsmässigt kontaktas per telefon av forskargruppen för att bjudas in att delta. Lämpliga studiedeltagare kommer att identifieras av personalen i den ordinarie verksamheten på de involverade vårdcentralerna med hjälp av screeningenkäter vid patientbesök eller telefon/-brevkontakt med patienter registrerade som tobaksbrukare i sina journaler. Lämpliga studiedeltagare kommer att innefatta vuxna (>18 år) dagliga tobaksanvändare. Vårdcentralerna kommer att tillhandahålla samtliga studiedeltagare behandling för tobaksavvänjning. Vilken behandling som studiedeltagarna får kommer att randomiseras på klusternivå. Detta

innebär att alla deltagare som rekryteras på en vårdcentral kommer att få samma behandling. Deltagarna kommer att tilldelas antingen ToR (definierat som rådgivande samtal i kombination med ett recept för individanpassad behandling för tobaksavvänjning, inklusive olika alternativ för rådgivning, läkemedel samt stöd till egenvård för tobaksavvänjning) eller standardbehandling. Behandlingsalternativen på receptet kommer att utgå från Socialstyrelsens riktlinjer för sjukdomsförebyggande metoder (8). Standardbehandlingen kommer att bestå av vårdcentralens nuvarande arbete med tobaksavvänjning. Som minsta insats krävs dock att enkla råd om tobaksavvänjning ges som standardbehandling. Samtliga studiedeltagare kommer att följas upp minst en gång efter det första behandlingstillfället. Då både interventions- och kontrollbehandlingen förväntas variera både inom och mellan grupperna kommer all tobaksbehandling som ges dokumenteras och i efterhand definieras. Samtliga behandlingar och insatser under studieperioden kommer av vårdpersonalen att dokumenteras i forskningsprotokoll/patientjournal för att senare kunna kvantifiera vårdkonsumtionen.

Effektiviteten av interventionen kommer att mätas genom att jämföra differensen i prevalens av tobaksanvändning (framgångsrika avvänjningsförsök), tobakskonsumtion (antal cigaretter per dag) samt hälsorelaterad livskvalitet före och efter interventionen (vid 6 och 12 månader). Powern att upptäcka en statistiskt signifikant skillnad ($p<0,05$) i andelen framgångsrika avvänjningsförsök mellan grupperna (14% i interventionsgruppen och 7% kontrollgruppen) kommer att vara åtminstone 63% baserat på en parad design med 7 vårdcentraler i interventionsgruppen och 7 vårdcentraler i kontrollgruppen som alla bidrar med minst 43 före- eftermätningar var, förutsatt en designeffekt på 1,5 på grund av "klustering" och 8% bortfall 6 månader efter interventionen. Data kommer att samlas in via enkäter samt genomgång av patientjournaler och relevanta register (såsom Läkemedelsregistret och det Gemensamma vårdregistret). Preliminära enkätfrågor finns redovisade i bilaga 5. Enkät 1 kommer att förmedlas till studiedeltagarna via personalen på vårdcentralen medan enkät 2 och 3 kommer att distribueras via post.

Involverad vårdpersonal kommer att utbildas och detaljerade studieprotokoll samt instruktioner för datainsamling kommer att utvecklas innan studiens start för att säkerställa datas tillförlitlighet. Forskargruppen kommer även att följa upp datainsamlingsprocessen och analysera insamlad data kontinuerligt för att säkerställa att upprättade rutiner efterföljs. Skillnader i före- och eftermätningarna kommer att analyseras baserat på statiska modeller. Faktorer som förbättrar utfall och riskfaktorer kommer också att analyseras. Demografiska och socioekonomiska karaktäristika samt hälsorelaterade beteenden kommer även att mätas i enkäterna för att kontrollera för ”confounders”. Direkta kostnader i form av utnyttjande av sjukvård, läkemedel, material och andra resurser kommer att samlas in genom forskningsprotokoll/patientjournaler och register för att i framtiden möjliggöra en hälsoekonomisk utvärdering av interventionen.

Studie II

I Studie II kommer en hälsoekonomisk utvärdering att genomföras, för att utvärdera kostnadseffektiviteten av ToR i jämförelse med befintliga strategier för tobaksprevention i primärvården, riktat mot socioekonomiskt utsatta grupper i Stockholms län. Analysen kommer att utföras ur ett livslångt perspektiv där även de framtida kostnaderna och konsekvenserna av jämförelsealternativen kommer att beaktas. För att kunna åstadkomma detta och integrera data från olika källor kommer en Markov-modell, särskilt framtagen för att utvärdera interventioner inom tobaksavvänjning, att tillämpas. Effektiviteten samt interventionskostnaderna kommer att baseras på data från Studie I. Ytterligare epidemiologisk data samt årlig medelkostnad per tobaksrelaterad sjukdom, kommer även att tas i beaktning i analysen. Dessa data kommer att samlas in från register samt rapporter och tidigare publicerade vetenskapliga artiklar. Kostnadseffektiviteten kommer att mätas som den inkrementella kostnaden per vunnet kvalitetsjusterat levnadsår (skillnaden i kostnaden, dividerat med, skillnaden i effektiviteten mellan jämförelsealternativen). Diskontering av kostnader och konsekvenser samt sensitivitetsanalys kommer att genomföras i enlighet med befintliga riktlinjer för hälsoekonomiska utvärderingar i Sverige.

Studie III

Vårdgivare som har förskrivit ToR och vuxna tobaksanvändare som förskrivits ToR i Studie I kommer av forskargruppen att rekryteras ändamålsmässigt till Studie III. Data kommer att samlas in genom semistrukturerade intervjuer i samtalsform, baserade på intervjuguider särskilt utvecklade för respektive målgrupp (klienter och vårdpersonal). För att öka validiteten kommer intervjuguiderna att pilottestas och vid behov justeras innan studiens start. Deltagarna kommer att tillfrågas om sina erfarenheter och synpunkter på ToR, inklusive upplevda förbättringsområden, fördelar och utmaningar med metoden samt viktiga aspekter för en eventuell framtida implementering av metoden. Preliminära intervjufrågor finns redovisade i bilaga 5. Kompletterande intervjuer kommer att genomföras med beslutsfattare för att fånga upp strukturella aspekter kring en eventuell implementering av metoden och förutsättningarna för denna typ av arbete i stort. Beslutsfattarnas deltagande förväntas bidra med ett helhetsperspektiv på metoden och verksamheten som den utvärderats i, samt en levande beskrivning av kontexten för Studie I-III. Intervjuerna kommer att genomföras på avtalad tid, i ett avskilt utrymme på vårdcentralen eller på annan plats utefter respondentens önskemål. Varje intervju beräknas pågå i cirka 30-60 minuter. Intervjuerna kommer att spelas in och transkriberas ordagrant, därefter kommer det manifesta innehållet i transkriptionerna att analyseras med hjälp av kvalitativ innehållsanalys, såsom beskrivits av Graneheim och Lundman (17). Data kommer att samlas tills mättnad uppnås eller tills inga fler nya mönster i svaren återfinns. Det förväntade antalet studiedeltagare är cirka 20-30 personer. Tillförlitligheten kommer att stärkas genom att ge studiedeltagarna möjlighet att kommentera resultatet och validera att forskargruppen förstått argumenten rätt.

Betydelse
Forskningen avser att generera kunskap om förskrivning av recept kan användas för att facilitera tobaksavvänjning i primärvården för socioekonomiskt utsatta grupper. Om så är fallet har ToR potential att bli en innovativ strategi för att åstadkomma livsstilsförändringar och förbättra folkhälsan i Sverige – inte minst i grupper som annars kan vara svåra att nå med hälsofrämjande insatser. ToR kan också ha en framtida praktisk betydelse i implementeringen av befintliga riktlinjer för sjukdomsförebyggande metoder, särskilt när det gäller primärvårdens arbete med tobaksavvänjning.

Preliminära resultat
Det finns inga preliminära resultat avseende effektiviteten och kostnadseffektiviteten ToR, men det finns vetenskaplig evidens för att FaR är en effektiv åtgärd som förbättrar hälsa och livskvalitet (14). Dessutom är FaR redan en etablerad del av arbetet med Socialstyrelsens riktlinjer för sjukdomsförebyggande metoder. Då själva receptet ännu inte är utformat finns inte heller några preliminära resultat avseende vårdpersonals och klienters upplevelser av att förskriva eller bli förskrivna receptet.

Etiska överväganden
Studiedeltagarna kommer att ges sedvanlig och evidensbaserad behandling för att uppnå tobaksavvänjning. Dock förväntas de i allmänhet söka vård för annat än tobaksavvänjning. Vid en sådan situation kan erbjudande om stöd för tobaksavvänjning uppfattas som negativt. En negativ upplevelse kan undvikas genom att förbereda klienten på att tobaksbruk kan bli aktuellt att diskutera, t.ex. genom att tillhandahålla förhandsinformation eller fråga klienten om det går bra att ta upp.

Trots många fördelar på både kort och lång sikt är det viktigt att ha i åtanke att tobaksavvänjning tillfälligt kan orsaka oönskat obehag och olika typer av abstinensbesvär hos klienten (18). Abstinensbesvär kan dock avhjälpas med förberedelser och med läkemedelsbehandling (18). Av Läkemedelsverket godkända nikotinersättningsmedel och/eller andra läkemedel för tobaksavvänjning (vareniklin, bupropion) kommer vid behov att erbjudas som en del av behandlingen. Vanliga biverkningar av nikotinersättningsmedel förekommer hos 10% av användarna och innefattar yrsel, huvudvärk och illamående (18). Trots tillförsel av nikotin är halterna i blodet lägre än vid tobaksbruk (18). Vanliga biverkningar av vareniklin innefattar illamående, onormala drömmar och sömnsvårigheter (18). Vanliga biverkningar av bupropion innefattar sömnsvårigheter, illamående och muntorrhet (18). Förekomsten av allvarliga biverkningar är <1% hos samtliga läkemedel som kan komma att ingå i studien (18–20). Trots viss risk för biverkningar är bruket kortvarigt och intaget av kemikalier mycket lägre vid läkemedelsbehandling än vid tobaksbruk, varför den förväntade nyttan överstiger riskerna och läkemedel med fördel kan förordas i avvänjningsprocessen.

Riskerna med eventuella biverkningar kommer även att minimeras genom förskrivning av dessa läkemedel i enlighet med föreskrifter samt Socialstyrelsens riktlinjer för sjukdomsförebyggande metoder (8). Förskrivande vårdpersonal kommer även att utbildas

i de behandlingsalternativ som studien avser. Vidare kommer uppföljning av studiedeltagarna att genomföras för att identifiera eventuella biverkningen av behandlingen. Studiedeltagarna kommer även uppmanas att uppsöka vård eller kontakta vårdcentralen och/eller sjukvårdsupplysningen om allvarliga eller långvariga biverkningar skulle uppstå. Vid eventuella biverkningar ska studiedeltagarna också informera vårdcentralen eller förskrivande vårdpersonal om dessa samt rådgöra om fortsatt behandling.

I övrigt finns det en risk för att datainsamlingen kan upplevas som påträngande då personliga och ibland känsliga erfarenheter behandlas (21). Både enkätfrågorna och intervjufrågorna kommer i förväg att pilottestas med representanter från respektive målgrupp (klienter och vårdpersonal) samt konsulteras med vår rådgivande kommitté som har mycket stor erfarenhet av liknande undersökningar. Detta för att säkerställa frågornas lämplighet gällande språk, innehåll, känslighet, tidsåtgång, etc. Studiedeltagarna kommer i Studie III att vara delaktiga i valet av tid och plats för datainsamlingen (intervju på avtalad tid i ett avskilt utrymme på vårdcentralen, alternativt på annan plats efter studiedeltagarens önskemål). Möjligheten att påverka förutsättningarna för intervjun kan förebygga negativa upplevelser av datainsamlingen. Studiedeltagarna kommer efter intervjun att tillfrågas om sin upplevelse av datainsamlingen. De kommer även få möjlighet att kommentera resultatet för att validera att forskargruppen förstått de angivna argumenten rätt.

På den sociala nivån, måste studiedeltagarnas förmåga att klara av påfrestningar tas i åtanke och det bör övervägas om det är lämpligt att utsätta en redan svag eller utsatt grupp för ytterligare påfrestningar (22). Av detta skäl finns det riktlinjer för att man inte bör forska på svaga eller utsatta grupper, om den kunskap man söker kan erhållas genom forskning på andra grupper av försökspersoner (22). Forskargruppens bedömning är dock att nyttan, behovet och kunskapsluckan är större vad gäller avvänjningsstöd till socioekonomiskt utsatta tobaksanvändare, jämfört med andra grupper. Vidare ska det finnas en klar nytta och minimerad risk för studiedeltagaren att delta i forskningen, särskilt om studiedeltagaren redan sedan tidigare är utsatt (22). Detta har tagits i åtanke i samtliga steg i processen och en klar nytta för studiedeltagarna ses då samtliga deltagare kommer att erbjudas någon typ av stöd för tobaksavvänjning som kan hjälpa dem att sluta använda tobak och bli friskare.

Vidare bör nämnas att sociala skillnader mellan forskargruppen och studiedeltagarna, såsom kön, ålder, socioekonomisk status, kulturell bakgrund, språk, m.m., kan bidra till att missförstånd eller feltolkningar i kommunikation, datainsamling och -analys kan uppstå (23). Forskargruppen är väl medveten om detta och kommer aktivt att arbeta för att förebygga detta i största möjliga mån.

Sist men inte minst bör nämnas att all tobaksanvändning hälsovådlig, oavsett socioekonomisk status. Tobaksavvänjning men även minskad tobakskonsumtion förbättrar hälsan och livskvaliteten hos individen och dess omgivning (6). Det leder även

till minskade kostnader för individ, sjukvård och samhälle (5). Stöd för tobaksavvänjning bör därför erbjudas alla som använder tobak. Detta projekt fokuserar dock på tobaksavvänjning i socioekonomiskt utsatta områden. Detta motiveras av ett ökat behov i målgruppen som har en högre förekomst av tobaksanvändning jämfört med den generella befolkningen (3). Socioekonomiskt utsatta grupper bör även prioriteras då dessa är svårare att nå med hälsofrämjande insatser (8). Trots att de flesta som besöker vårdcentralerna som är involverade i studien förväntas ha en lägre socioekonomisk status kommer kriterier för socioekonomisk status inte att tillämpas i rekryteringen p.g.a. etiska skäl. Socioekonomisk status kommer dock att dokumenteras för att beskriva studiedeltagarna samt kontrollera för eventuell "confounding". Skulle ToR visa sig vara en effektiv metod för tobaksavvänjning i primärvården bör ToR vid en framtida implementering dock erbjudas samtliga tobaksanvändare, då det gynnar alla tobaksanvändare att sluta oavsett socioekonomisk status.

Referenser

1. World Health Organization. WHO REPORT on the global TOBACCO epidemic, 2008 The MPOWER package. 2008.
2. Socialstyrelsen. Registeruppgifter om tobaksrökningens skadeverkningar. 2014.
3. Galanti MR, Gilljam H, Post A, Eriksson B. Tobaksbruk i länet. 2011.
4. Lyons R, Lo SV, Littlepage B. Perception of Health Amongst Ever-Smokers and Never-smokers. Tob Control. 1994;3:213–5.
5. Bolin K, Borgman B, Gip C, Wilson K. Current and future avoidable cost of smoking--estimates for Sweden 2007. Health Policy. 2011 Nov;103(1):83–91.
6. U.S. Department of Health and Human Services. The Health Consequences of Smoking—50 Years of Progress A Report of the Surgeon General. Atlanta; 2014.
7. Reinfeldt F, Larsson M. Regeringens proposition 2007/08:110 En förnyad folkhälsopolitik. Regeringskansliet; 2007.
8. Socialstyrelsen. Nationella riktlinjer för sjukdomsförebyggande metoder 2011. Tobaksbruk, riskbruk av alkohol, otillräcklig fysisk aktivitet och ohälsosamma matvanor. Stöd för styrning och ledning. 2011.
9. Statens Folkhälsoinstitut. På väg mot ett tobaksfritt landsting - En uppföljning av landstingens och regionernas policyarbete kring tobaksprevention 2009. Växjö; 2010.
10. Hiscock R, Bauld L, Amos A, Fidler JA, Munafò M. Socioeconomic status and smoking: a review. Ann N Y Acad Sci. 2012 Feb;1248:107–23.
11. Regionala cancercentrum i samverkan. RCC:s handlingsplan för ett rökfritt Sverige. 2014.

12. Walander A, Ålander S, Burström B. Sociala skillnader i vårdutnyttjande. Stockholm; 2004.

13. Leppänen A, Biermann O, Sundberg CJ, Tomson T. Perceived feasibility of a primary care intervention for Tobacco Cessation on Prescription targeting disadvantaged groups in Sweden – a qualitative study (submitted). 2015.

14. Kallings L. Physical activity on prescription : Studies on physical activity level, adherence and cardiovascular risk factors. PhD thesis. Karolinska Institutet; 2008.

15. Hjalmarson A, Attebring MF, Herlitz J. Svårt implementera avvänjning från tobak i ordinarie vårdrutin. Läkartidningen. 2012;109(26-28):1290–3.

16. Burström B, Walander A, Viberg I, Bruce D, Agerholm J, Ponce de Leon A. Förslag till behovsindex 2011-2013. Stockholm; 2013.

17. Graneheim UH, Lundman B. Qualitative content analysis in nursing research: concepts, procedures and measures to achieve trustworthiness. Nurse Educ Today. 2004 Feb;24(2):105–12.

18. Holm Ivarsson B, Hjalmarson A, Pantzar M. Stödja patienter att sluta röka och snusa. Lund: Studentlitteratur; 2012.

19. FASS. Champix Produktinformation [Internet]. Hämtad 5 februari från: http://www.fass.se/LIF/product?1&userType=2&nplId=20060105000057&docTyp e=6

20. FASS. Zyban Produktinformation [Internet]. Hämtad 5 februari från: http://www.fass.se/LIF/product?4&userType=2&nplId=20000519000014&docTyp e=6

21. Bäärnhielm S, Ekblad S. Qualitative Research, Culture and Ethics: A Case Discussion. Transcult Psychiatry. 2002;39(4):469–83.

22. Forskningsetisk policy och organisation i Sverige. Riktlinjer för etisk värdering av medicinsk humanforskning. Uppsala; 2003.

23. Green J, Thorogood N. Qualitative Methods for Health Research . Andra upplagan. London: Sage Publications Limited; 2009.
