# Supplementary material for: Motivation 2 Quit (M2Q): A cluster randomized controlled trial evaluating the effectiveness of Tobacco Cessation on Prescription in Swedish primary healthcare
Source: PLoS One. 2022 Dec 1;17(12):e0278369. doi: 10.1371/journal.pone.0278369 (PMC9714827; doi:10.1371/journal.pone.0278369)
Supplement: S3 File — (PDF) [file pone.0278369.s003.pdf]

Appendix 2

Research plan for Tobacco Cessation on Prescription as a primary healthcare intervention in disadvantaged areas in Stockholm County

Scientific research questions

The overall aim of this project is to explore whether a prescription approach can be used to facilitate tobacco cessation among socioeconomically disadvantaged groups in Swedish primary healthcare. This will be achieved by answering the following research questions:

1. What is the effectiveness of TCP compared to standard treatment for tobacco cessation in Swedish primary healthcare? (Study I)

2. What is the cost-effectiveness of TCP compared to standard treatment for tobacco cessation in Swedish primary healthcare? (Study II)

3. What are the perceived barriers and facilitators of implementing TCP as a primary healthcare intervention in socioeconomically disadvantaged areas in Sweden? (Study III)

Overview of the field

Tobacco use is seen as the leading cause of death in the world (1) and estimated to cause approximately 10% of all deaths in Sweden (2). Furthermore, chronic disease caused by tobacco use is a large public health problem in Sweden – particularly in socioeconomically disadvantaged groups where the prevalence of tobacco use is markedly higher than in the general population (3). In addition to the negative impact of tobacco use on the health and quality of life of the population (4), it is also associated with increased costs for the healthcare system but also for society at large (5). Tobacco cessation has been found to reduce the risk of premature disease and death from tobacco-related diseases (6), wherefore this is a prioritized area in Swedish public health policy (7). In Sweden, the National Board of Health and Welfare has issued national guidelines for disease prevention methods, where treatment for tobacco cessation is included (8). This includes treatments such as brief advice, counseling (in combination with nicotine replacement therapy or targeted follow-up), qualified counseling (in combination with nicotine replacement therapy, varenicline or bupropion), proactive telephone counseling, as well as web and computer-based counseling (8). The guidelines recommend that healthcare providers should offer all tobacco users support to quit (8). Despite this, the treatment intensity for tobacco cessation is relatively low (9) and high-risk groups with a greater need for support are often difficult to reach (8). The difficulties could be explained by a lower social support, lower motivation to quit, higher tobacco dependance, higher likelihood to discontinue treatment and support for behavioral change, as well as psychological differences like increased susceptibility to marketing from the tobacco industry (10). The need for a more structured approach to tobacco cessation treatment in healthcare and better access to cessation support for socioeconomically disadvantaged groups was recently emphasized (11).

Studies conducted on healthcare consumption in different social groups in Stockholm show that individuals with foreign origin, low educational level and lower income often turn to primary

healthcare (12). The public has great trust in the healthcare system and most persons who use tobacco seek care for different health problems in primary healthcare centers. Thus, primary healthcare has a significant potential to support tobacco cessation efforts.

In a recent pilot study, the perceived acceptance, feasibility, as well as benefits and disadvantages of using Tobacco Cessation on Prescription (TCP) as a tool for health promotion for socioeconomically disadvantaged groups in primary healthcare in Stockholm, were explored (13). The results were based on semi-structured interviews with clients, healthcare providers and experts on other lifestyle interventions on prescription (13). In addition to requests on content and design of TCP, the study found that the tool could have a positive emotional meaning for clients (e.g. concerning motivation and perceived ”right” to seek care for tobacco cessation), while it could have a positive practical meaning for healthcare providers (facilitating planning, documentation, etc.) (13).

The idea is that TCP in the future could be implemented and prescribed in a similar manner as Physical Activity on Prescription (PAP), another lifestyle intervention on prescription that has been shown to improve health and quality of life and that is already used to prevent chronic disease in Sweden (14). Lifestyle interventions on prescription can lead to patients taking advice more seriously when these are given by a person with the authority of a doctor/healthcare provider. In addition, the tendency to change behavior can increase if clear advice is given and prescribed with the same dedication and conviction as pharmacotherapy (15). TCP can therefore be seen as a potential tool to facilitate and structure tobacco cessation treatment and tobacco prevention in primary healthcare according to existing guidelines (13). Despite expected benefits, a prescription approach to tobacco cessation has previously not been evaluated.

Project description

The project will consist of three sub-studies that are intended to be conducted in 2015-2019 as part of a PhD project at the Department of Learning, Informatics, Management and Ethics at Karolinska Institutet. The project is funded by the Stockholm County Council.

Study I

Approximately 654 study participants will be recruited from 14 primary healthcare centers in socioeconomically disadvantaged areas in Stockholm. Eligible primary healthcare centers will be identified by the research group based on a socioeconomic index (16) and purposefully contacted by telephone by the research group to be invited to participate. Eligible study participants will be identified by the staff members as part of the standard practice at the involved primary healthcare centers, either through screening questionnaires during patient visits or telephone/mail contact with patients that are registered as tobacco users in their electronic medical records. Eligible study participants will include adult (> 18 years) daily tobacco users. The primary healthcare centers will offer all the study participants treatment for tobacco cessation. Which treatment the study participants receive will be randomized at cluster level. This means that all participants that are recruited at one primary healthcare center will receive the same treatment. The participants will receive either TCP (defined as counseling

combined with a prescription for individualized treatment for tobacco cessation, including different options for counseling, pharmacotherapy and support for self-management for tobacco cessation) or standard treatment. The treatment options on the prescription form will be based on the National Board of Health and Welfare's guidelines for disease prevention methods (8). Standard treatment will consist of the primary healthcare centers' current practice for tobacco cessation treatment. However, the minimum requirement is that brief advice is given as standard treatment. All study participants will be followed up at least once after the first visit. Since the treatment in both the intervention and control group is expected to vary both within and between the treatment arms, all tobacco cessation treatment provided will be documented and defined in retrospect. All treatments and measures during the study period will be documented in study protocols/the electronic medical record by the healthcare providers to later enable quantification of the healthcare consumption.

The effectiveness of the intervention will be measures by comparing the difference in prevalence of tobacco use (successful quit attempts), tobacco consumption (number of cigarettes per day) and health-related quality of life before and after the intervention (at 6 and 12 months). The power to detect a statistically significant difference ($p<0{,}05$) in the proportion of successful quit attempts between the groups (14% in the intervention group and 7% in the control group) will be at least 63% based on a paired design with 7 primary healthcare centers in the intervention group and 7 primary healthcare centers in the control group that all contribute with at least 43 before and after measurements each, considering a design effect of 1.5 due to clustering and 8% drop-out at 6 months after the intervention. Data will be collected through questionnaires and review of electronic medical records and relevant registers (the Drug register and Common healthcare register). Preliminary questionnaire items are provided in Appendix 5. Questionnaire 1 will be distributed to the study participants by staff members at the primary healthcare centers, while questionnaires 2 and 3 will be distributed via mail.

Involved primary healthcare providers will be trained and detailed study protocols, as well as instructions for data collection will be developed before the start of the study to ensure reliability of data. The research group will also follow up the data collection procedure and review collected data continuously to ensure that the established routines are followed. Differences in before and after measures will be analyzed based on statistical models. Factors that improve outcomes and risk factors will also be analyzed. Demographic and socioeconomic characteristics, as well as health-related behaviors will also be measured in the questionnaires to control for confounders. Direct costs in the form of healthcare consumption, pharmacotherapy, materials and other resources will be collected through research protocols/electronic medical records and registers to enable a future health economic evaluation of the intervention.

Study II

In Study II, a health economic evaluation will be conducted to evaluate the cost-effectiveness of TCP compared to standard treatment for tobacco cessation in primary healthcare with a focus on socioeconomically disadvantaged groups in Stockholm County. The analysis will be conducted with a lifetime time horizon where the future costs and consequences of the treatment

alternatives also will be considered. To achieve this and to integrate data from various sources a Markov model, specifically designed to evaluate tobacco cessation interventions, will be applied. The effectiveness and intervention costs will be based on data from Study I. Additional epidemiological data and average annual cost per tobacco-related disease will also be included in the analysis. This data will be collected from registers, reports and previously published scientific articles. The cost-effectiveness will be measured as the incremental cost per quality-adjusted life year (difference in cost, divided by the difference in effectiveness between the treatment alternatives). Discounting of costs and consequences, as well as sensitivity analysis, will be conducted according to best practice guidelines for health economic evaluations in Sweden.

Study III

Healthcare providers that have prescribed TCP and adult tobacco users that have been prescribed TCP in Study I, will be purposefully recruited by the research group to participate in Study III. Data will be collected through semi-structured interviews in conversational form, based on interview guides specifically developed for each target group (clients and healthcare providers). To increase the validity, the interview guides will be pilot tested and adjusted if needed before the start of the study. The participants will be asked about their experiences of and opinions on TCP, including perceived areas for improvement, benefits and challenges with the intervention and important aspects for a potential implementation of the intervention. Preliminary interview questions are provided in Appendix 5. Complementary interviews will be conducted with decision makers to capture structural aspects regarding a potential implementation of the intervention and the prerequisites for this type of treatment in general. The participation of the decision makers is expected to contribute to a holistic perspective on the intervention and the setting in which it is evaluated, as well as an evocative description of the context for Study I-III. The interviews will be conducted on a scheduled time in a private space at the primary healthcare center or another location based on the participants' preferences. Each interview is expected to be approximately 30-60 minutes long. The interviews will be recorded and transcribed verbatim, thereafter the manifest content of the transcripts will be analyzed based on qualitative content analysis, as described by Graneheim and Lundman (17). Data will be collected until saturation is achieved or until no more emerging patterns in the responses can be found. The expected number of study participants is approximately 20-30. The trustworthiness will be strengthened by giving the study participants opportunity to comment on the results and validate whether the research group has understood their accounts correctly.

<u>Significance</u>

The research intends to generate knowledge on whether a prescription approach can be used to facilitate tobacco cessation in primary healthcare among socioeconomically disadvantaged groups. If that is the case, TCP has the potential to become an innovative strategy to achieve lifestyle changes and improve public health in Sweden – not least in groups that otherwise are difficult to reach with health promotion efforts. TCP may also have a future practical role in implementing existing guidelines for disease prevention methods, particularly when it comes to tobacco cessation treatment in primary healthcare.

Preliminary results

There are no preliminary results regarding the effectiveness and cost-effectiveness of TCP but there is scientific evidence that PAP is an effective intervention that improves health and quality of life (14). In addition, PAP is already an established part of the National Board of Health and Welfare's guidelines on disease prevention methods. Since the prescription form itself has not been designed yet, there are no preliminary results regarding healthcare providers' or clients' experiences of prescribing or being prescribed TCP.

Ethical considerations

The study participants will be given conventional and evidence-based treatment to achieve tobacco cessation. However, they are generally expected to seek care for other health problems than tobacco cessation. To be offered support for tobacco cessation in this situation may be perceived negatively. A negative experience could be avoided by preparing the client that tobacco use may be discussed, e.g. by providing information about the study in advance or by asking the client if this is acceptable to bring up for discussion.

Even though there are many advantages with tobacco cessation in the short and long term, it is important to consider that tobacco cessation can cause unwanted discomfort and different types of withdrawal symptoms among clients (18). However, withdrawal symptoms could be avoided with preparations and pharmacotherapy (18). Nicotine replacement therapies and/or other pharmaceuticals for tobacco cessation (varenicline, bupropion) that are approved by the Medical Products Agency, will be offered as part of the treatment if needed. Common side effects of nicotine replacement therapy occur among 10% of users and include dizziness, headache and nausea (18). Although nicotine is administered, the level of nicotine in the blood is lower during nicotine replacement therapy than during tobacco use (18). Common side effects of varenicline include nausea, abnormal dreams and sleeping difficulties (18). Common side effects of bupropion include sleeping difficulties, nausea and dryness of the mouth (18). The prevalence of serious side effects is <1% among all pharmacotherapies that may be included in the study (18-20). Despite some risk for side effects, the treatment period is relatively short and the intake of chemicals much lower during pharmacotherapy than during tobacco use, wherefore the benefit is expected to outweigh the risks and pharmaceuticals can advantageously be used in the cessation process.

The risks of potential side effects will also be minimized by prescribing these pharmaceuticals in accordance with instructions and the National Board of Health and Welfare's guidelines for disease prevention methods (8). Responsible staff members will also be trained in the treatment options included in the study. In addition, the study participants will be followed up to identify potential side effects of the treatment. The study participants will also be encouraged to seek care or contact the primary healthcare center and/or healthcare services if serious or long-term side effects occur. If side effects occur, the study participants should inform the primary healthcare center or responsible staff members about this and discuss continued treatment.

Furthermore, there is a risk that the data collection can be perceived as intrusive since personal and sometimes sensitive experiences are explored (21). Both the questionnaire items and

interview questions will be pilot tested in advance with representatives from the target group (clients and healthcare providers) and discussed with our expert advisory committee that has extensive experience of similar studies. This is to ensure the appropriateness of the questions regarding language, content, sensitivity, time, etc. The study participants in Study III will be involved in the choice of time and location of the data collection (interview at a scheduled time in a private space at the primary healthcare center, or another location according to the participant's preference). The possibility to influence the prerequisites for the interview may prevent a negative experience of the data collection. After the interview, the study participants will be asked about their experiences of the data collection. They will also be given the opportunity to comment on the results and validate that the research group has understood their accounts correctly.

At the social level, the study participants' ability to handle strain must be taken into account and it should be considered whether it is appropriate to expose an already vulnerable population to further strain (22). For this reason, there are guidelines saying that research on vulnerable populations should be avoided if the knowledge could be obtained by conducting research on other populations (22). However, the research group's assessment is that the benefit, the need and the knowledge gap is greater when it comes to cessation support for socioeconomically disadvantaged tobacco users, compared to other groups. In addition, there should be a clear benefit and minimized risk for study participants to take part in research, particularly if they are already at risk (22). This has been taken into consideration in each step of the research process and a clear benefit for the study participants is seen since all participants will be offered some type of support for tobacco cessation that could help them to quit their tobacco use and become healthier.

Furthermore, it should be noted that social differences between the research group and the study participants, such as gender, age, socioeconomic status, cultural background, language, etc, could contribute to misunderstandings or misinterpretations in communication, data collection and analysis (23). The research group is very well aware of this and will work actively to prevent this as much as possible.

Last but not least, it should be noted that all tobacco use is hazardous for health, irrespective of socioeconomic status. Tobacco cessation, but also decreased tobacco use, improves health and quality of life among individuals and their environment (6). It also leads to reduced costs for the individual, the healthcare system and society (5). Treatment for tobacco cessation should therefore be offered to all tobacco users. However, this project focuses on tobacco cessation in socioeconomically disadvantaged areas. This is motivated by a greater need in this target group that has a higher prevalence of tobacco use compared to the general population (3). Socioeconomically disadvantaged groups should also be prioritized since they are more difficult to reach with health promotion efforts (8). Although most of those who visit the primary healthcare centers that are involved in the study are expected to have a lower socioeconomic status, eligibility criteria for this will not be applied due to ethical reasons. However, data on socioeconomic status will be collected to describe the study participants and control for potential confounding. If TCP is shown to be an effective intervention for tobacco

cessation in primary healthcare, TCP should if implemented in the future, be offered to all tobacco users since it benefits all tobacco users to quit, irrespective of their socioeconomic status.

References

1. World Health Organization. WHO REPORT on the global TOBACCO epidemic, 2008 The MPOWER package. 2008.

2. Socialstyrelsen. Registeruppgifter om tobaksrökningens skadeverkningar. 2014.

3. Galanti MR, Gilljam H, Post A, Eriksson B. Tobaksbruk i länet. 2011.

4. Lyons R, Lo SV, Littlepage B. Perception of Health Amongst Ever-Smokers and Never-Smokers. Tob Control. 1994;3:213-5.

5. Bolin K, Borgman B, Gip C, Wilson K. Current and future avoidable cost of smoking – estimates for Sweden 2007. Health Policy. 2011 Nov; 103(1):83-91.

6. U.S. Department of Health and Human Services. The Health Consequences of Smoking – 50 Years of Progress A Report of the Surgeon General. Atlanta; 2014.

7. Reinfeldt F, Larsson M. Regeringens proposition 2007/08:110 En förnyad folkhälsopolitik. Regeringskansliet; 2007.

8. Socialstyrelsen. Nationella riktlinjer för sjukdomsförebyggande metoder 2011. Tobaksbruk, riskbruk av 7lcohol, otillräcklig fysisk aktivitet och ohälsosamma matvanor. Stöd för styrning och ledning. 2011.

9. Statens Folkhälsoinstitut. På väg mot ett tobaksfritt landsting – En uppföljning av landstingens och regionernas policyarbete kring tobaksprevention 2009. Växjö; 2010.

10. Hiscock R, Bauld L, Amos A, Fidler JA, Munafo M. Socioeconomic status and smoking: a review. Ann N Y Acad Sci. 2012 Feb;1248:107-23.

11. Regionala cancercentrum i samverkan. RCC:s handlingsplan för ett rökfritt Sverige. 2014.

12. Walander A, Ålander S, Burström B. Sociala skillnader i vårdutnyttjande. Stockholm; 2004.

13. Leppänen A, Biermann O, Sundberg CJ, Tomson T. Perceived feasibility of a primary care intervention for Tobacco Cessation on Prescription targeting disadvantaged groups in Sweden – a qualitative study (submitted). 2015.

14. Kallings L. Physical activity on prescription : Studies on physical activity level, adherence and cardiovascular risk factors. PhD thesis. Karolinska Institutet; 2008.

15. Hjalmarsson A, Attebring MF, Herlitz J. Svårt implementera avvänjning från tobak i ordinarie vårdrutin. Läkartidningen. 2012;109(26-28):1290-3.

16. Burström B, Walander A, Viberg I, Bruce D, Agerholm J, Ponce de Leon A. Förslag till behovsindex 2011-2013. Stockholm; 2013.

17. Graneheim UH, Lundman B. Qualitative content analysis in nursing research: concepts, procedures and measures to achieve trustworthiness. Nurse Educ Today. 2004 Feb;24(2):105-12.

18. Holm Ivarsson B, Hjalmarsson A, Pantzar M. Stödja patienter att sluta röka och snusa. Lund: Studentlitteratur; 2012.

19. FASS. Champix Produktinformation [Internet]. Accessed February 5th from: http://www.fass.se/LIF/product?1&userType=2&nplId=20060105000057&docType=6

20. FASS. Zyban Produktinformation [Internet]. Accessed February 5th from: http://www.fass.se/LIF/product?4&userType=2&nplId=20000519000014&docType=6

21. Bäärnhielm S, Ekblad S. Qualitative Research, Culture and Ethics: A Case Discussion. Transcult Psychiatry. 2002;39(4):469-83.

22. Forskningsetisk policy och organisation i Sverige. Riktlinjer för etisk värdering av medicinsk humanforskning. Uppsala; 2003.

23. Green J, Thorogood N. Qualitative Methods for Health Research. 2nd edition. London: Sage Publications Limited; 2009.
