# Supplementary material for: Motivation 2 Quit (M2Q): A cluster randomized controlled trial evaluating the effectiveness of Tobacco Cessation on Prescription in Swedish primary healthcare
Source: PLoS One. 2022 Dec 1;17(12):e0278369. doi: 10.1371/journal.pone.0278369 (PMC9714827; doi:10.1371/journal.pone.0278369)
Supplement: S4 File — (DOCX) [file pone.0278369.s004.docx]

**Table 1. 7-day abstinence at 6 months follow-up unadjusted.**

|  | | OR (95% CI) | SE |
| --- | --- | --- | --- |
| Treatment | |  |  |
|  | Intervention | 3.54 (1.06-11.90) | 2.19 |
| Intercept | | 0.15 (0.05-0.44) | 0.08 |
|  | | ICC (95% CI) | SE |
|  | | 0.028 (1.3e-04-0.87) | 0.08 |

**Table 2. 7-day abstinence at 6 months follow-up adjusted.**

|  | | OR (95% CI) | SE | |
| --- | --- | --- | --- | --- |
| Treatment | |  |  | |
|  | Intervention | 5.45 (1.57-18.93) | 3.46 | |
| Gender | |  |  | |
|  | Female | 2.65 (1.09-6.47) | 1.21 | |
| Intention to quit | |  |  | |
|  | Time undefined | 0.07 (0.01-0.63) | 0.08 | |
|  | >6 months | 0.19 (0.02-1.78) | 0.22 | |
|  | Within 2-6 months | 0.38 (0.14-1.01) | 0.19 | |
| PHC operation | |  |  | |
|  | Private | 0.26 (0.11-0.65) | 0.12 | |
| Intercept | | 0.18 (0.05-0.63) | 0.11 | |
|  | | ICC (95% CI) | SE | |
|  | | 2.1e-07 (1.7e-67-1.00) | | 1.5e-05 |

**Table 3. 3-month abstinence at 6 months follow-up unadjusted.**

|  | | OR (95% CI) | SE | |
| --- | --- | --- | --- | --- |
| Treatment | |  |  | |
|  | Intervention | 3.38 (0.96-11.98) | 2.18 | |
| Intercept | | 0.11 (0.03-0.35) | 0.07 | |
|  | | ICC (95% CI) | SE | |
|  | | 9.4e-07 (2.7e-51-1.00) | | 4.9e-05 |

**Table 4. 3-month abstinence at 6 months follow-up adjusted.**

|  | | OR (95% CI) | | SE | |
| --- | --- | --- | --- | --- | --- |
| Treatment | |  | |  | |
|  | Intervention | 6.36 (1.30-31.27) | | 5.17 | |
| Gender | |  | |  | |
|  | Female | 4.08 (1.34-12.45) | | 2.32 | |
| Intention to quit | |  | |  | |
|  | Time undefined | 0.10 (0.01-0.89) | | 0.11 | |
|  | >6 months | 0.25 (0.03-2.33) | | 0.28 | |
|  | Within 2-6 months | 0.27 (0.09-0.80) | | 0.15 | |
| PHC operation | |  | |  | |
|  | Private | 0.18 (0.05-0.70) | | 0.13 | |
| PHC number of listed patients | | 1.00 (1.00-1.00) | 1.4e-04 | | |
| PHC number of employees | | 0.95 (0.88-1.02) | | 0.04 | |
| Intercept | | 0.08 (0.02-0.41) | | 0.07 | |
|  | | ICC (95% CI) | | SE | |
|  | | 3.4e-08 (2.9e-52-1.00) | | | 1.8e-06 |

**Table 5. Any cigarette quit attempt at 6 months follow-up unadjusted.**

|  | | OR (95% CI) | SE |
| --- | --- | --- | --- |
| Treatment | |  |  |
|  | Intervention | 1.49 (0.46-4.85) | 0.90 |
| Intercept | | 0.98 (0.36-2.68) | 0.50 |
|  | | ICC (95% CI) | SE |
|  | | 0.069 (3.2e-03-0.63) | 0.10 |

**Table 6. Any cigarette quit attempt at 6 months follow-up adjusted.**

|  | | OR (95% CI) | SE |
| --- | --- | --- | --- |
| Treatment | |  |  |
|  | Intervention | 1.14 (0.40-3.23) | 0.61 |
| Previous quit attempts | |  |  |
|  | 1 | 2.65 (0.43-16.31) | 2.46 |
|  | 2-3 | 3.60 (0.91-14.26) | 2.53 |
|  | 4-5 | 5.59 (1.12-27.81) | 4.57 |
|  | >6 | 5.27 (1.56-17.85) | 3.28 |
| Intercept | | 0.33 (0.09-1.18) | 0.22 |
|  | | ICC (95% CI) | SE |
|  | | 0.011 (3.0e-08-1.00) | 0.07 |

**Table 7. Cigarettes per day among non-quitters at 6 months follow-up unadjusted.**

|  | | Coefficient (p-value) | SE |
| --- | --- | --- | --- |
| Treatment | |  |  |
|  | Intervention | 0.54 (0.754) | 1.73 |
| Intercept | | 11.37 (0.000) | 1.47 |
|  | | ICC (95% CI) |  |
|  | | 3.0e-24 (3.0e-24-3.0e-24) | 0.00 |

**Table 8. Cigarettes per day among non-quitters at 6 months follow-up adjusted.**

|  | | Coefficient (p-value) | SE |
| --- | --- | --- | --- |
| Treatment | |  |  |
|  | Intervention | 0.54 (0.754) | 1.73 |
| Intercept | | 11.37 (0.000) | 1.47 |
|  | | ICC (95% CI) | SE |
|  | | 3.0e-24 (3.0-e24-3.0e-24) | 0.00 |

**Table 9. Change in EQ-5D index at 6 months follow-up unadjusted.**

|  | | Coefficient (p-value) | | SE |
| --- | --- | --- | --- | --- |
| Treatment | |  | |  |
|  | Intervention | -0.02 (0.547) | | 0.04 |
| Intercept | | 0.01 (0.860) | | 0.03 |
|  | | ICC (95% CI) | | SE |
|  | | | 5.1e-25 (5.1e-25-5.1e-25) | 0.00 |

**Table 10. Change in EQ-5D index at 6 months follow-up adjusted.**

|  | | Coefficient (p-value) | | SE |
| --- | --- | --- | --- | --- |
| Treatment | |  | |  |
|  | Intervention | 3.6e-03 (0.919) | | 0.03 |
| Intention to quit | |  | |  |
|  | > 6 months | -0.16 (0.034) | | 0.08 |
|  | Within 2-6 months | -1.9e-03 (0.972) | | 0.05 |
|  | Within 1 month | 0.03 (0.513) | | 0.05 |
| Importance to quit | |  | |  |
|  | Moderate | -0.28 (0.008) | | 0.11 |
|  | High | -0.32 (0.001) | | 0.10 |
| Intercept | | 0.29 (0.004) | | 0.10 |
|  | | | ICC (95% CI) | SE |
|  | | | 4.2e-24 (4.2e-24-4.2e-24) | 0.00 |

**Table 11. 7-day abstinence at 12 months follow-up unadjusted.**

|  | | OR (95% CI) | SE |
| --- | --- | --- | --- |
| Treatment | |  |  |
|  | Intervention | 3.38 (1.03-11.15) | 2.06 |
| Intercept | | 0.16 (0.05-0.49) | 0.09 |
|  | | ICC (95% CI) | SE |
|  | | 0.025 (6.2e-04-0.51) | 0.05 |

**Table 12. 7-day abstinence at 12 months follow-up adjusted.**

|  | | OR (95% CI) | SE | |
| --- | --- | --- | --- | --- |
| Treatment | |  |  | |
|  | Intervention | 3.35 (0.98-11.45) | 2.10 | |
| Gender | |  |  | |
|  | Female | 2.93 (1.28-6.98) | 1.30 | |
| Intention to quit | |  |  | |
|  | Time undefined | 0.05 (0.01-0.46) | 0.06 | |
|  | >6 months | 0.17 (0.02-1.60) | 0.19 | |
|  | Within 2-6 months | 0.41 (0.16-1.04) | 0.20 | |
| PHC operation | |  |  | |
|  | Private | 0.27 (0.11-0.67) | 0.12 | |
| Intercept | | 0.28 (0.07-1.06) | 0.19 | |
|  | | ICC (95% CI) | SE | |
|  | | 4.2e-07 (2.8e-49-1.00) | | 2.1e-05 |

**Table 13. 3-month abstinence at 12 months follow-up unadjusted.**

|  | | OR (95% CI) | SE |
| --- | --- | --- | --- |
| Treatment | |  |  |
|  | Intervention | 5.71 (1.27-25.81) | 4.40 |
| Intercept | | 0.08 (0.02-0.34) | 0.06 |
|  | | ICC (95% CI) | SE |
|  | | 3.7e-03 (4.3e-13-1.00) | 0.04 |

**Table 14. 3-month abstinence at 12 months follow-up adjusted.**

|  | | | OR (95% CI) | SE | |
| --- | --- | --- | --- | --- | --- |
| Treatment | | |  |  | |
|  | Intervention | | 7.80 (1.25-48.82) | 7.30 | |
| Gender | | |  |  | |
|  | Female | | 4.66 (1.62-13.38) | 2.51 | |
| Intention to quit | | |  |  | |
|  | Time undefined | | 0.07 (0.01-0.65) | 0.08 | |
|  | >6 months | | 0.26 (0.03-2.45) | 0.30 | |
|  | Within 2-6 months | | 0.45 (0.17-1.24) | 0.23 | |
| PHC operation | | |  |  | |
|  | Private | | 0.26 (0.09-0.74) | 0.14 | |
| PHC socioeconomic index | | | 1.01 (0.99-1.02) | 0.01 | |
| Intercept | | 3.8e-03 (4.5e-06-3.21) | | 0.01 | |
|  | | | ICC (95% CI) | SE | |
|  | | | 9.4e-08 (3.3e-50-1.00) | | 4.7e-06 |

**Table 15. Any cigarette quit attempt at 12 months follow-up unadjusted.**

|  | | OR (95% CI) | SE | |
| --- | --- | --- | --- | --- |
| Treatment | |  |  | |
|  | Intervention | 1.01 (0.40-2.53) | 0.47 | |
| Intercept | | 0.85 (0.38-1.89) | 0.35 | |
|  | | ICC (95% CI) | SE | |
|  | | 1.5e-09 (0-∞) | | 10.0e-06 |

**Table 16. Any cigarette quit attempt at 12 months follow-up adjusted.**

|  | | OR (95% CI) | SE | |
| --- | --- | --- | --- | --- |
| Treatment | |  |  | |
|  | Intervention | 0.70 (0.25-1.92) | 0.36 | |
| Importance to quit | |  |  | |
|  | Moderate | 0.17 (0.03-0.85) | 0.14 | |
|  | High | (omitted due to collinearity) |  | |
| Intercept | | 1.43 (0.57-3.61) | 0.67 | |
|  | | ICC (95% CI) | SE | |
|  | | 3.6e-08 (0-∞) | | 3.4e-05 |

**Table 17. Cigarettes per day among non-quitters at 12 months follow-up unadjusted.**

|  | | Coefficient (p-value) | SE |
| --- | --- | --- | --- |
| Treatment | |  |  |
|  | Intervention | 0.25 (0.879) | 1.64 |
| Intercept | | 10.61 (0.000) | 1.41 |
|  | | ICC (95% CI) | SE |
|  | | 4.2e-20 (4.2e-20-4.2e-20) | 0.00 |

**Table 18. Cigarettes per day among non-quitters at 12 months follow-up adjusted.**

|  | | | Coefficient (p-value) | SE |
| --- | --- | --- | --- | --- |
| Treatment | | |  |  |
|  | Intervention | -0.57 (0.757) | | 1.84 |
| Heaviness of Smoking Index | | |  |  |
|  | Moderate | | 2.04 (0.264) | 1.83 |
|  | High | | 7.72 (0.000) | 2.17 |
| Ever-use of pharmacotherapy | | |  |  |
|  | Yes | | 2.51 (0.100) | 1.51 |
| Intercept | | | 6.55 (0.001) | 2.04 |
|  | | | ICC (95% CI) | SE |
|  | | | 0.090 (5.1e-03-0.65) | 0.12 |

**Table 19. Change in EQ-5D index at 12 months follow-up unadjusted.**

|  | | | Coefficient (p-value) | SE |
| --- | --- | --- | --- | --- |
| Treatment | | |  |  |
|  | Intervention | -0.02 (0.781) | | 0.06 |
| Intercept | | | 0.01 (0.829) | 0.05 |
|  | | | ICC (95% CI) | SE |
|  | | | 0.022 (6.8e-04-0.43) | 0.04 |

**Table 20. Change in EQ-5D index at 12 months follow-up adjusted.**

|  | | | Coefficient (p-value) | SE |
| --- | --- | --- | --- | --- |
| Treatment | | |  |  |
|  | Intervention | 4.5e-04 (0.994) | | 0.06 |
| Importance to quit | | |  |  |
|  | Moderate | | -0.16 (0.355) | 0.17 |
|  | High | | -0.29 (0.077) | 0.16 |
| Intercept | | | 0.27 (0.100) | 0.16 |
|  | | | ICC (95% CI) | SE |
|  | | | 0.024 (9.7e-04-0.38) | 0.04 |
