# Supplementary material for: Motivation 2 Quit (M2Q): A cluster randomized controlled trial evaluating the effectiveness of Tobacco Cessation on Prescription in Swedish primary healthcare
Source: PLoS One. 2022 Dec 1;17(12):e0278369. doi: 10.1371/journal.pone.0278369 (PMC9714827; doi:10.1371/journal.pone.0278369)
Supplement: S5 File — (DOCX) [file pone.0278369.s005.docx]

**Table 1. 7-day abstinence at 6 months follow-up unadjusted.**

|  | | Coefficient (95% CI) | SE |
| --- | --- | --- | --- |
| Treatment | |  |  |
|  | Intervention | 1.27 (0.05-2.48) | 0.62 |
| Intercept | | -1.92 (-3.02- -0.82) | 0.56 |

**Table 2. 7-day abstinence at 6 months follow-up adjusted.**

|  | | Coefficient (95% CI) | SE |
| --- | --- | --- | --- |
| Treatment | |  |  |
|  | Intervention | 1.70 (0.45-2.94) | 0.64 |
| Gender | |  |  |
|  | Female | 0.97 (0.08-1.87) | 0.46 |
| Intention to quit | |  |  |
|  | Time undefined | -2.64 (-4.82- -0.46) | 1.11 |
|  | >6 months | -1.65 (-3.88-0.58) | 1.14 |
|  | Within 2-6 months | -0.96 (-1.93-0.01) | 0.50 |
| PHC operation | |  |  |
|  | Private | -1.33 (-2.24- -0.43) | 0.46 |
| Intercept | | -1.70 (-2.94- -0.47) | 0.63 |

**Table 3. 3-month abstinence at 6 months follow-up unadjusted.**

|  | | Coefficient (95% CI) | SE |
| --- | --- | --- | --- |
| Treatment | |  |  |
|  | Intervention | 1.22 (-0.05-2.48) | 0.64 |
| Intercept | | -2.23 (-3.42- -1.04) | 0.61 |

**Table 4. 3-month abstinence at 6 months follow-up adjusted.**

|  | | Coefficient (95% CI) | | SE |
| --- | --- | --- | --- | --- |
| Treatment | |  | |  |
|  | Intervention | 1.85 (0.26-3.44) | | 0.81 |
| Gender | |  | |  |
|  | Female | 1.41 (0.29-2.52) | | 0.57 |
| Intention to quit | |  | |  |
|  | Time undefined | -2.32 (-4.53- -0.12) | | 1.12 |
|  | >6 months | -1.40 (-3.65-0.85) | | 1.15 |
|  | Within 2-6 months | -1.33 (-2.44- -0.22) | | 0.57 |
| PHC operation | |  | |  |
|  | Private | -1.69 (-3.02- -0.36) | | 0.68 |
| PHC number of listed patients | | 1.8e-04 (-9.3e-05-4.6e-04) | 1.4e-04 | |
| PHC number of employees | | -0.05 (-0.13-0.02) | | 0.04 |
| Intercept | | -2.50 (-4.08- -0.90) | | 0.81 |

**Table 5. Any cigarette quit attempt at 6 months follow-up unadjusted.**

|  | | Coefficient (95% CI) | SE |
| --- | --- | --- | --- |
| Treatment | |  |  |
|  | Intervention | 0.40 (-0.79-1.58) | 0.60 |
| Intercept | | -0.02 (-1.03-0.99) | 0.51 |

**Table 6. Any cigarette quit attempt at 6 months follow-up adjusted.**

|  | | Coefficient (95% CI) | SE |
| --- | --- | --- | --- |
| Treatment | |  |  |
|  | Intervention | 0.13 (-0.91-1.17) | 0.53 |
| Previous quit attempts | |  |  |
|  | 1 | 0.98 (-0.84-2.79) | 0.93 |
|  | 2-3 | 1.28 (-0.10-2.66) | 0.70 |
|  | 4-5 | 1.72 (0.12-3.33) | 0.82 |
|  | >6 | 1.66 (0.44-2.88) | 0.62 |
| Intercept | | -1.10 (-2.37-0.17) | 0.65 |

**Table 7. 7-day abstinence at 12 months follow-up unadjusted.**

|  | | Coefficient (95% CI) | SE |
| --- | --- | --- | --- |
| Treatment | |  |  |
|  | Intervention | 1.22 (0.03-2.41) | 0.61 |
| Intercept | | -1.81 (-2.90- -0.71) | 0.56 |

**Table 8. 7-day abstinence at 12 months follow-up adjusted.**

|  | | Coefficient (95% CI) | SE |
| --- | --- | --- | --- |
| Treatment | |  |  |
|  | Intervention | 1.21 (-0.02-2.44) | 0.63 |
| Gender | |  |  |
|  | Female | 1.07 (0.20-1.94) | 0.44 |
| Intention to quit | |  |  |
|  | Time undefined | -2.93 (-5.09- -0.77) | 1.10 |
|  | >6 months | -1.78 (-4.02-0.47) | 1.15 |
|  | Within 2-6 months | -0.90 (-1.84-0.04) | 0.48 |
| PHC operation | |  |  |
|  | Private | -1.30 (-2.20- -0.41) | 0.46 |
| Intercept | | -1.27 (-2.59-0.06) | 0.68 |

**Table 9. 3-month abstinence at 12 months follow-up unadjusted.**

|  | | Coefficient (95% CI) | SE |
| --- | --- | --- | --- |
| Treatment | |  |  |
|  | Intervention | 1.74 (0.24-3.25) | 0.77 |
| Intercept | | -2.53 (-3.97- -1.08) | 0.74 |

**Table 10. 3-month abstinence at 12 months follow-up adjusted.**

|  | | | Coefficient (95% CI) | SE |
| --- | --- | --- | --- | --- |
| Treatment | | |  |  |
|  | Intervention | | 2.05 (0.22-3.89) | 0.94 |
| Gender | | |  |  |
|  | Female | | 1.54 (0.48-2.59) | 0.54 |
| Intention to quit | | |  |  |
|  | Time undefined | | -2.60 (-4.76- -0.43) | 1.11 |
|  | >6 months | | -1.35 (-3.60-0.90) | 1.15 |
|  | Within 2-6 months | | -0.79 (-1.80-0.22) | 0.51 |
| PHC operation | | |  |  |
|  | Private | | -1.36 (-2.41- -0.31) | 0.54 |
| PHC socioeconomic index | | | 0.01 (-0.01-0.02) | 0.01 |
| Intercept | | -5.58 (-12.32-1.17) | | 3.44 |

**Table 11. Any cigarette quit attempt at 12 months follow-up unadjusted.**

|  | | Coefficient (95% CI) | SE |
| --- | --- | --- | --- |
| Treatment | |  |  |
|  | Intervention | 0.01 (-0.91-0.93) | 0.47 |
| Intercept | | -0.17 (-0.97-0.64) | 0.41 |

**Table 12. Any cigarette quit attempt at 12 months follow-up adjusted.**

|  | | Coefficient (95% CI) | SE |
| --- | --- | --- | --- |
| Treatment | |  |  |
|  | Intervention | -0.36 (-1.38-0.65) | 0.52 |
| Importance to quit | |  |  |
|  | Moderate | -1.77 (-3.38- -0.16) | 0.82 |
|  | High | (omitted due to collinearity) |  |
| Intercept | | 0.36 (-0.56-1.28) | 0.47 |
